# Differences in cerebral small vessel disease magnetic resonance imaging markers between lacunar stroke and non–Lobar intracerebral hemorrhage

**DOI:** 10.1177/23969873211031753

**Published:** 2021-08-25

**Authors:** Kim Wiegertjes, Michelle G Jansen, Wilmar MT Jolink, Marco Duering, Emma A Koemans, Floris HBM Schreuder, Anil M Tuladhar, Marieke JH Wermer, Catharina JM Klijn, Frank-Erik de Leeuw

**Affiliations:** 1 Department of Neurology, Donders Institute for Brain, Cognition and Behavior, Radboud University Medical Center, Nijmegen, The Netherlands; 2 Department of Neurology and Neurosurgery, University Medical Center Utrecht, Brain Center, Utrecht University, Utrecht, The Netherlands; 3 Institute for Stroke and Dementia Research, LMU University Hospital Munich, Munich, Germany; 4 Munich Cluster for Systems Neurology, Munich, Germany; 5 Department of Neurology, Leiden University Medical Center, Leiden, The Netherlands

**Keywords:** Magnetic resonance imaging, small vessel disease, stroke, intracerebral hemorrhage, lacunar stroke

## Abstract

**Introduction:**

It is unclear why cerebral small vessel disease (SVD) leads to lacunar stroke in some and to non–lobar intracerebral hemorrhage (ICH) in others. We investigated differences in MRI markers of SVD in patients with lacunar stroke or non–lobar ICH.

**Patients and methods:**

We included patients from two prospective cohort studies with either lacunar stroke (RUN DMC) or non–lobar ICH (FETCH). Differences in SVD markers (white matter hyperintensities [WMH], lacunes, cerebral microbleeds [CMB]) between groups were investigated with univariable tests; multivariable logistic regression analysis, adjusted for age, sex, and vascular risk factors; spatial correlation analysis and voxel–wise lesion symptom mapping.

**Results:**

We included 82 patients with lacunar stroke (median age 63, IQR 57–72) and 54 with non-lobar ICH (66, 59–75). WMH volumes and distribution were not different between groups. Lacunes were more frequent in patients with a lacunar stroke (44% vs. 17%, adjusted odds ratio [aOR] 5.69, 95% CI [1.66–22.75]) compared to patients with a non–lobar ICH. CMB were more frequent in patients with a non–lobar ICH (71% vs. 23%, aOR for lacunar stroke vs non–lobar ICH 0.08 95% CI [0.02–0.26]), and more often located in non–lobar regions compared to CMB in lacunar stroke.

**Discussion:**

Although we obserd different types of MRI markers of SVD within the same patient, ischemic markers of SVD were more frequent in the ischemic type of lacunar stroke, and hemorrhagic markers were more prevalent in the hemorrhagic phenotype of non-lobar ICH.

**Conclusion:**

There are differences between MRI markers of SVD between patients with a lacunar stroke and those with a non-lobar ICH.

## Introduction

Cerebral small vessel disease (SVD) is the presumed underlying cause of up to 25% of all ischemic strokes and 85% of intracerebral hemorrhages (ICH).^1,2^ SVD refers to pathological changes of the small vessels of the brain, and can manifest itself in hereditary and sporadic forms.^3^ Cerebral amyloid angiopathy (CAA) primarily affects the superficial perforating arteries, whereas the non–CAA form of SVD mainly affects the deep perforating arteries.^3^

It is still poorly understood why some patients with the similar form of non-CAA sporadic SVD present with an ischemic lacunar stroke whereas others present with a non-lobar ICH.^4,5^

Consequences of SVD can be visualized by its markers on magnetic resonance imaging (MRI), including white matter hyperintensities (WMH), lacunes, and cerebral microbleeds (CMB).^6^ Previously, periventricular WMH burden has been associated with a lacunar stroke, whereas presence of CMB was associated with a non–lobar ICH.^7^ This study, however, did not investigate differences in spatial distribution patterns of SVD markers on MRI between groups, which could be essential for understanding the clinical course of SVD. Our results may potentially identify MRI markers of SVD that differentially predispose to either ischemia or hemorrhage, that in time may have implications for secondary preventive treatment.

Therefore, we aimed to investigate whether presence and spatial distribution of WMH, lacunes, and CMB on MRI differ between patients with a lacunar stroke and those with a non–lobar ICH.

## Patients and methods

### Study population

We identified patients from two prospective cohort studies; individuals with a lacunar stroke from the Radboud University Nijmegen Diffusion tensor and MRI Cohort (RUN DMC) and with a non–lobar ICH from the Finding the ETiology in spontaneous Cerebral Hemorrhage (FETCH) study. The Medical Review Ethics Committee Region Arnhem-Nijmegen approved the RUN DMC study and the medical ethics committee of the UMCU approved the FETCH study. All patients gave written informed consent.

The RUN DMC is a single–center, prospective, cohort study which investigated 503 non–demented elderly, aged between 50–85 years old, with evidence of SVD on MRI (WMH or lacunes).^8^ All participants underwent a structural interview, clinical assessment, and a 1.5 Tesla (T) MRI protocol. In this study, we included patients with lacunar stroke (ischemic stroke or transient ischemic attack [TIA]) in their medical history (Supplementary Figure 1). Patients were excluded if there was evidence for any other presumed cause of ischemia in their medical history (i.e. including large artery disease, cardioembolic source, or embolic stroke or TIA of undetermined source) or if they had a history of ICH. If neuroimaging was available in the patients’ file, subcortical MRI lesions consistent with clinical symptoms were used as confirmation that clinical symptoms were caused by lacunar infarction.

The FETCH study is a multi–center, prospective, cohort study amongst 204 adults that presented with a symptomatic spontaneous ICH confirmed by computed tomography (CT).^9^ Patients underwent 3 T and/or 7 T MRI in one of three participating centers (University Medical Centers of Utrecht, Leiden or Nijmegen). Secondary causes were excluded by CT angiography in all and by digital subtraction angiogram if clinically indicated. For this study, we included patients of 50 years and older with a 3 T MRI and ICH in the basal ganglia, thalamus, brainstem or cerebellum.

Demographics and vascular risk factors were assessed and defined as follows: age at the time of the MRI; sex; hypertension as the use of antihypertensive medication, systolic blood pressure ≥140 mm Hg, or diastolic blood pressure ≥90 mm Hg, based on the average of three (RUN DMC) or two (FETCH; in medical history before the ICH) blood pressure measurements or left ventricular hypertrophy on ECG; diabetes mellitus as the usage of oral antidiabetics and/or insulin (RUN DMC) or as reported in medical history and/or two fasting glucose measurements of >7 mmol/l (FETCH); history of smoking as ever or never smoked; alcohol overuse as alcohol use ≥300 g per week; and body mass index (BMI) by dividing the weight in kilograms by the height in meters squared.

### MRI markers of SVD

#### MRI data

In the RUN DMC study, participants were scanned using one single 1.5 T MRI scanner (Siemens Healthineers, Erlangen, Germany), whereas the FETCH study used three different 3 T MRI scanners (Siemens Healthineers, Erlangen, Germany; Phillips Healthcare, Best, The Netherlands). For a detailed overview of the MRI sequences of both studies (and participating centers) please see Supplementary Table 1. We rated WMH, lacunes, and CMB in accordance with the STandards for ReportIng Vascular ChangEs on neuroimaging (STRIVE) criteria.^6^

#### White matter hyperintensities

WMH segmentation on FLAIR sequences was performed as previously published for the RUN DMC dataset,^10^ and segmented in the ICH-free hemisphere for the FETCH dataset, using intensity–based thresholding in MRIcro (https://www.mricro.com), and subsequent manual adjustment by one of two trained raters. The ICH-free hemisphere was used because WMH cannot be distinguished from perihematomal edema, if located in neighboring areas. WMH volumes were expressed as a percentage of intracranial volume in both datasets.

WMH masks were normalized to Montreal Neurological Institute (MNI) 152 standard space using the Functional Magnetic Resonance Imaging of the Brain Software Library Software Library (FSL).^11^ First, we skull–stripped each image using the FSL Brain Extraction Tool (BET). Second, we registered FLAIR to T1 images using the FSL Linear Image registration tool (FLIRT; correlation ratio). Third, we used FLIRT and the FSL Non–linear Registration Tool (FNIRT) to register T1 images to the MNI template. Lastly, we applied the resulting transformation matrices to the WMH masks. We generated bilateral masks in the FETCH dataset by inverting WMH masks and registering them to the contralateral hemisphere. All registration steps were checked visually. We generated WMH frequency maps displaying the proportion of participants with WMH in any given voxel for visualization purposes.

### Lacunes

Lacunes were assessed by location by one of two trained raters, and categorized as lobar (centrum semiovale, frontal, parietal, insular/subinsular, temporal, occipital) or non–lobar (basal ganglia, thalamus, internal and external capsule, brain stem, cerebellum).^12^ Agreement with a second rater in random subsamples was good (Cohen’s kappa > 0.7 in both datasets). Final decisions were made in consensus involving more experienced raters (FEdL, MD). All lacunes were manually segmented on T1 images, and normalized to MNI 152 standard space via T1 images using the registration tools ‘FLIRT’ and ‘FNIRT’ from FSL.^11^ We created spherical maps, with each sphere representing a single lacune, to visualize the distribution of lacunes over lobar and non–lobar regions.

### Microbleeds

CMB were assessed by one trained rater (RUN DMC) or screened by a semiautomatic method after which true CMB were selected by a human rater (FETCH).^13^ Location of CMB was determined by using the Microbleed Anatomical Rating Scale (MARS),^14^ categorizing distribution as lobar (frontal, parietal, temporal, occipital, insula) and non–lobar (basal ganglia, thalamus, internal and external capsule, corpus callosum, deep and periventricular white matter, brain stem, cerebellum). A second rater assessed microbleeds in a random subsample (Cohen’s kappa ≥ = 0.70 in both datasets), indicating good inter–rater agreement. Final consensus was reached during meetings with experienced clinical neurologists (FEdL, FHBMS). After manual segmentation, CMB lesion masks were normalized to MNI 152 standard space via T1 images, and spherical maps were created with each sphere representing one single CMB.

### Statistical data analysis

All analyses were performed using R (version 3.5.3; https://www.R–project.org). We considered two–tailed p values <0.05 to be statistically significant. Demographics, vascular risk factors, and MRI markers of SVD were compared between patients with a lacunar stroke and a non–lobar ICH using univariable tests (independent sample *t* test, Chi–squared test, and Mann–Whitney U test where appropriate). We investigated associations of SVD MRI markers with either of the two stroke types using an age– and sex adjusted multivariable logistic regression model including vascular risk factors that were significant in univariate tests (glm *R* package). Additionally, for patients with ≥1 lacune or ≥1 CMB, we compared the percentage of lobar vs non–lobar lesions using non–parametric univariate tests (Mann–Whitney U tests).

To compare differences in WMH distribution between groups, we performed a spatial correlation analysis and voxel–wise lesion symptom mapping (VLSM). For each voxel in MNI 152 standard space, the lesion frequency was compared between groups using linear correlation, where a correlation coefficient of 1 indicates an identical lesion distribution. Furthermore, we investigated whether a voxel in standard space more frequently was a WMH (yes/no) in lacunar stroke or non–lobar ICH using VLSM in in NiiStat (https://www.nitrc.org/projects/niistat/). We only included voxels that were damaged in at least 4% of the patients,^15^ and adjusted analysis for age and total WMH volume. Permutation–based thresholding was used to control for family–wise error (FWE) at 5% (*p* < .05, two–tailed, 5000 Freedman–Lane permutations).

## Results

In total, we included 82 patients with a lacunar stroke (63% males, median age 63 years, interquartile range [IQR] 57–72, 45 ischemic stroke and 37 TIA). Additionally, 54 patients with a non–lobar ICH were included (74% males, median age 66 years, IQR 59–75; Supplementary Figure 1, 35 deep and 19 infratentorial). Eight of 54 (15%) patients had more than one ICH. The median stroke–MRI interval for patients with lacunar stroke was 198 days (IQR 95–630), and for patients with non–lobar ICH 13.5 days (6–38). Patient characteristics are summarized in Table 1. Patients with lacunar stroke more frequently had hypertension and a history of smoking than those with non–lobar ICH (Table 1).

**Table 1.** Cohort characteristics and associations with lacunar stroke and non-lobar intracerebral hemorrhage.

	Lacunar stroke (N = 82)	Non-lobar ICH (N = 54)	Mean differences (95% CI)	Univariable OR (95% CI)	p-Value	Multivariable OR (95% CI)	p-Value
Demographics							
Age at MRI, years, median [IQR]	63 [57–72]	66 [59–75]	2.6 (–1.1; 6.6)		.198	0.95 (0.89; 1.01)	.092
Male sex, N (%)	52 (63%)	39 (73%)		0.7 (0.3; 1.5)	.286	3.52 (1.26; 10.35)	**.018**
Vascular risk factors							
Hypertension, N (%)	66 (80%)	32 (60%)		2.8 (1.3; 6.2)	**.012**	6.72 (2.11; 24.76)	**.002**
Diabetes mellitus, N (%)	15 (18%)	8 (15%)		1.3 (0.5; 3.4)	.597		
History of smoking, N (%)	69 (84%)	30 (57%)		4.1 (1.8; 9.3)	**<.001**	3.38 (1.13; 10.71)	**.032**
Alcohol overuse, N (%)	27 (36%)	15 (29%)		1.4 (0.6; 3.0)	.429		
BMI, kg/m^2^, mean (SD)	27 ± 4	25 ± 5	–1.2 (–2.8; 0.4)		.111		
MRI markers of SVD							
WMH volume, % ICV, median [IQR]	0.3 [0.1–0.8]	0.4 [0.2–0.9]	0.07 (–0.05; 0.2)		.227		
Presence of lacunes, N (%)	36 (44%)	9 (17%)		3.7 (1.7–9.1)	**.001**	5.69 (1.66; 22.75)	**.008**
Presence of CMB, N (%)	19 (23%)	35 (67%)		0.2 (0.1; 0.3)	**<.001**	0.08 (0.02; 0.26)	**<.001**
ICV, mL, mean (SD)	1484 (146)	1476 (166)	–7.7 (–67; 52)		.969		

ICH: intracerebral hemorrhage; CI: confidence interval; OR: odds ratio; IQR: interquartile range; SD: standard deviation; BMI: body mass index; WMH: white matter hyperintensities; CMB: cerebral microbleeds; ICV: intracranial volume. Values represent median [IQR], N (%), or mean (SD). Information on history of smoking was missing in 1 (1%), alcohol overuse in 8 (6%), WMH volumes in 9 (7%), presence of lacunes in 2 (1%), CMB presence in 3 (2%), and ICV in 5 (4%) patients. Multivariable odd ratios and corresponding 95% confidence intervals represent results from the logistic regression analysis for lacunar stroke versus non-lobar intracerebral hemorrhage (ICH) adjusted for age, sex, and vascular risk factors significant in univariate analysis.p-values (the significant ones are printed in bold).

### MRI markers of SVD

#### White matter hyperintensities

**Figure 1.**
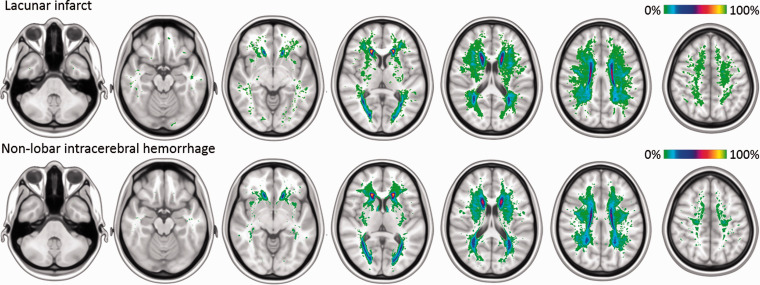
Distribution of white matter hyperintensities in patients with lacunar stroke or non–lobar intracerebral hemorrhage. Frequency maps of white matter hyperintensities (WMH) superimposed on a MNI–152 0.5 mm template, where each voxel represents the percentage of individuals with a WMH in that voxel, as indicated by color–coded bars.

The median WMH volume was similar in patients with a lacunar stroke (0.3 mL IQR [0.1–0.8] and a non–lobar ICH (0.4 mL [0.2–0.9], *p*=.227). WMH were most prevalent in the periventricular and frontal white matter in both groups (Figure 1). The spatial correlation analysis demonstrated that the colocalization of WMH between groups was strong (*R*^2^=0.71; *p* < 2.2e–16). VSLM analysis, adjusted for age, sex, and normalized WMH volume, showed a comparable distribution of WMH as there were no major clusters of voxels with WMH associated with either a lacunar stroke or a non–lobar ICH (Supplementary Figure 2).

#### Lacunes

**Figure 2.**
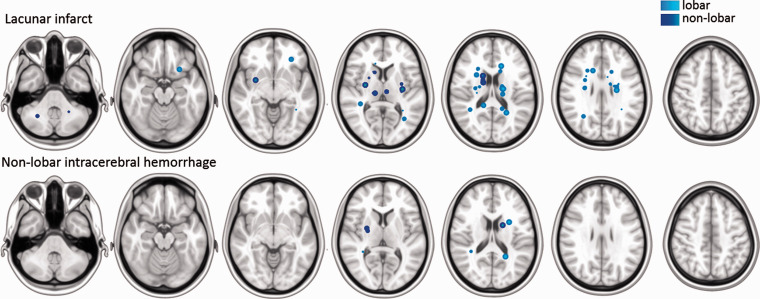
Distribution of lacunes in patients with lacunar stroke or non–lobar intracerebral hemorrhage. Spherical maps of lacunes superimposed on a MNI–152 0.5 mm template with each sphere indicating a single lacune, colour–coding represents lobar (light blue) or non–lobar (dark blue) locations.

Of 82 patients with a lacunar stroke, 36 (44%) had at least one lacune, in comparison with 9 of 54 patients with a non–lobar ICH (17%, *p*=.001). Presence of lacunes was significantly associated with a lacunar stroke after adjustment for age, sex, history of hypertension and smoking (aOR 5.69, 95% CI 1.66–22.75, Table 1). In patients with a lacunar stroke, 51 of 70 lacunes (73%) were in lobar regions, and 8 of 10 (80%) in patients with a non–lobar ICH (*p* = 0.423, Table 2, Figure 2).

**Table 2.** Lesion counts by lobar and non-lobar brain regions in patients with lacunar stroke or non-lobar intracerebral hemorrhage, with at least one lacune or cerebral microbleed.

	Lacunar stroke	Non-lobar ICH	p-Value
Lacunes, N
Total	70	10	
Lobar	51 (73%)	8 (80%)	.423
Non-Lobar	19 (27%)	2 (20%)	.206
CMB, N
Total	76	302	
Lobar	59 (78%)	162 (54%)	.006
Non-Lobar	17 (22%)	140 (46%)	.008

ICH: intracerebral hemorrhage; CMB: cerebral microbleeds. Values represent N (%). Lesion counts were compared between groups using univariate Mann–Whitney U tests.

#### Cerebral microbleeds

**Figure 3.**
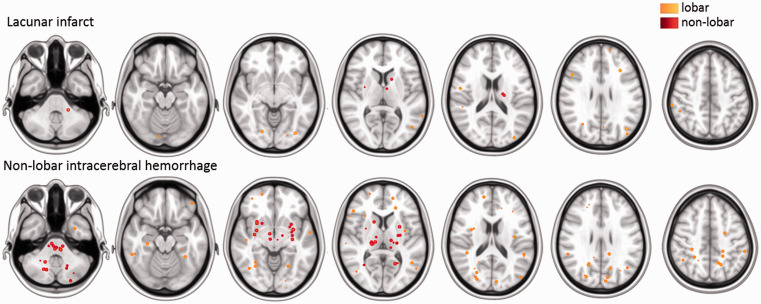
Distribution of cerebral microbleeds in patients with lacunar stroke or non–lobar intracerebral hemorrhage. Spherical maps of cerebral microbleeds superimposed on a MNI–152 0.5 mm template with each sphere indicating a single microbleed, colour–coding represents lobar (orange) or non–lobar (red) locations.

Of the patients with a lacunar stroke, 19 (23%) had CMB, in comparison with 35 of patients with a non–lobar ICH (67%, *p* < .001). Presence of CMB was significantly associated with a non–lobar ICH after adjustment for age, sex, history of hypertension and smoking (aOR for lacunar stroke vs non–lobar ICH 0.08, 95% CI 0.02–0.26). In patients with a lacunar stroke, 59 of 76 CMB (78%) were in lobar regions, and in patients with a non–lobar ICH 162 of 302 CMB (54%) were in lobar regions (*p*=.006, Table 2, Figure 3).

## Discussion

Patients with a lacunar stroke and patients with a non–lobar ICH have similar volume and spatial distribution of WMH. Lacunes were more frequent in patients with a lacunar stroke, and CMB were more prevalent in patients with a non–lobar ICH. Spatial distribution of lacunes was similar in patients with a lacunar stroke and a non–lobar ICH, but CMB were more often non–lobar in patients with a non–lobar ICH compared to CMB in patients with a lacunar stroke.

In contrast to our findings of a similar burden and distribution of WMH, in a previous study severe confluent periventricular WMH were found to be associated with a lacunar stroke compared to a non–lobar ICH.^7^ However, these findings were based on visual ratings of WMH, whereas in our study we made use of quantitative measures and a voxel–based approach. In our study, WMH were most frequent in periventricular areas, around the anterior and posterior horns of the lateral ventricles (Figure 1). The periventricular regions are known to be specifically vulnerable to ischemia,^16,17^ as they are located at the arterial end zone (the very ends of arterial territories) including the junction of the deep and superficial perforating arteries.^18,19^ Previous data demonstrated a strong inverse voxel–wise correlation between resting–state perfusion and WMH frequency, in individuals with CAA, mild cognitive impairment, Alzheimer’s disease and healthy controls,^20^ indicating that across these different populations, WMH are most frequent in regions with relatively lower cerebral perfusion. However, this needs to be confirmed in prospective studies.

Even though far more frequent in lacunar stroke, patients with a non–lobar ICH also had lacunes. Alterations in cerebral hemodynamics, blood–brain barrier permeability, release of inflammatory cytokines, and blood pressure after an ICH may give rise to a higher frequency of ischemic lesions.^21^ Likewise, the incidence of CMB has been reported to be relatively high after ischemic stroke.^22^ In addition to a similar distribution of WMH, patients with a lacunar stroke or a non–lobar ICH showed a large similarity in the distribution of lacunes, suggesting a common pathophysiological mechanism. This could be explained by previous studies within genetically defined SVD, where incident lacunes were found to predominantly occur within the orientation of perforating arteries,^23^ and the majority were localized at the edges of WMH.^24^ Another study demonstrated that lobar lacunes were in contact with WMH in 80% of the cases and were highly correlated with WMH volume, suggesting a common origin.^12^ This might explain the he high proportion of lobar lacunes found in our study, as WMH are predominantly located in lobar white matter regions, such as the centrum semiovale.

In contrast, CMB were more often lobar in patients with a lacunar stroke compared to patients with a non–lobar ICH. The high proportion of CMB in lobar brain regions in subcortical SVD remains unexplained. We are not able to fully exclude the possibility that CAA might have contributed to the high proportion of lobar CMB. Another study found evidence of moderate to severe CAA in around 6 of 48 (13%) participants with non-lobar ICH (i.e. arteriosclerotic subcortical small vessel disease).^25^ Furthermore, deep CMB were found to be mainly associated with arteriosclerotic small vessel disease, whereas both CAA and arteriosclerotic small vessel disease contributed to the risk of lobar CMB.^26^ Also, cerebellar hemorrhages might be due to CAA if located in superficial regions.^27^ Collectively, these results suggest that arteriosclerotic SVD and CAA often co-exist, possibly resulting in a higher rate of lobar CMB.

SVD can manifest itself as ischemic or hemorrhagic disease, between which many risk factors are shared. If we gain more insight into the pathophysiological mechanisms that determine whether someone is more prone to ischemic or hemorrhagic disease, this could inform treatment decisions. In clinical practice, antiplatelets are prescribed in patients with a lacunar stroke but not after a non–lobar ICH, as antiplatelet therapy was considered to increase the risk of ICH.^28^ However, the recently completed RESTART clinical trial found survivors of antithrombotic–associated ICH to have fewer recurrences of ICH when antiplatelet therapy was restarted compared to patients in whom antiplatelet therapy was avoided.^29^ In addition, in contrast with previous suggestions, cumulative evidence suggests that presence of CMB should not be a reason to refrain from antiplatelet therapy. A recent pooled analysis of individual patient data of patients with ischemia stroke or TIA, showed that although presence of CMB enhance the risk of ICH to a larger extent than that of ischemic stroke, the absolute risk of ischemic stroke in these patients is higher than the absolute risk of ICH.^30^

Strengths of our study are the use of two prospectively collected cohorts, the meticulous phenotyping, and the combination of multiple statistical approaches, including hypothesis–free voxel–based methods. Our study also has limitations. First, as the two etiologies originated from different studies, this might have led to a systematic bias. For example, patients with a lacunar stroke had a 1.5 T MRI, which may have led to underestimation of SVD burden.^31^Although the impact of MRI field strength on WMH volume measurements besides the expected improved resolution is still unclear, a previous study in patients with multiple sclerosis demonstrated a 10% higher mean WMH volume using 3 T MRI versus 1.5 T MRI.^32^ Although there are no studies investigating the variability of lacunes according to MRI field strength, more CMB are found at higher MRI field strengths.^33^ For instance, a previous study in 25 patients with multiple sclerosis, 53 CMB were found using 3 T MRI compared to 41 CMB using 1.5 T MRI.^34^ Although the number of CMB increases with field strength and resolution,^31^ the detection of whether any CMB are present or not, generally remains consistent across different field strengths. Overall, the severity of MRI markers of SVD might have been underestimated on 1.5 T MRI scans. In addition, different blood-sensitive MRI sequences were used (SWI or T2*–weighted imaging), which also affects the number of CMB detected.^35^ Moreover, the timing of the MRI was different between datasets. Whereas FETCH patients were scanned in the acute phase, MRI was performed in the chronic phase in RUN DMC. Furthermore, differences in patient selection could have influenced our results. In the RUN DMC study, patients were selected based on the presence of MRI markers of SVD, which might have resulted in an overestimation of the prevalence of vascular risk factors and neuroimaging markers. Although both studies had a history of hypertension or hypertensive treatment as part of their definition, diagnosis of hypertension on the basis of blood pressure measurements slightly differed between studies. In the non-lobar ICH group this relied upon two independent blood pressure measurements (systolic blood pressure ≥140 mm Hg or diastolic blood pressure ≥90 mm Hg) reported in the medical history and did not include data obtained during clinical admission. Therefore, individuals in whom hypertension was discovered during follow up after the ICH, were not included in this definition. This may in part explain the lower prevalence of hypertension in the group of non-lobar ICH. Hypertension in the RUN DMC was based on blood pressure measurements (systolic blood pressure ≥140 mm Hg or diastolic blood pressure ≥90 mm Hg) at the time of inclusion. Furthermore, the finding that there were no differences in the prevalence of diabetes mellitus between groups might be due to the fact that the RUN DMC did not include fasting glucose measurements obtained during clinical admission, as we know from previous studies that diabetes mellitus is strongly associated with lacunar stroke compared to ICH. Second, the sample size was relatively small. Third, more severe ICHs are often fatal, and therefore less likely to be investigated by MRI, which may have resulted in an underestimation of burden of SVD markers on MRI. Fourth, we did not investigate the full spectrum of MRI markers of SVD. Future studies should investigate the role of other MRI markers of SVD, such as perivascular spaces or cortical microinfarcts, in differentiating patients with non-lobar ICH or lacunar infarcts.

In conclusion, SVD in patients with lacunar stroke and non–lobar ICH cannot be distinguished by WMH burden or distribution. Patients presenting with the ischemic phenotype of lacunar stroke more often had lacunes, whereas patients with the hemorrhagic phenotype of non–lobar ICH more frequently had CMB, which were more often non–lobar than CMB in patients with lacunar stroke. Future longitudinal studies in early disease stages should address the temporal order and location of the occurrence of ischemic and hemorrhagic lesions to elucidate the mechanisms through which SVD causes different lesion types.

## Supplemental Material

sj-pdf-1-eso-10.1177_23969873211031753 - Supplemental material for Differences in cerebral small vessel disease magnetic resonance imaging markers between lacunar stroke and non–Lobar intracerebral hemorrhage Click here for additional data file. Supplemental material, sj-pdf-1-eso-10.1177_23969873211031753 for Differences in cerebral small vessel disease magnetic resonance imaging markers between lacunar stroke and non–Lobar intracerebral hemorrhage by Kim Wiegertjes, Michelle G Jansen, Wilmar MT Jolink, Marco Duering, Emma A Koemans, Floris HBM Schreuder, Anil M Tuladhar, Marieke JH Wermer, Catharina JM Klijn and Frank-Erik de Leeuw in European Stroke Journal

sj-pdf-2-eso-10.1177_23969873211031753 - Supplemental material for Differences in cerebral small vessel disease magnetic resonance imaging markers between lacunar stroke and non–Lobar intracerebral hemorrhage Click here for additional data file. Supplemental material, sj-pdf-2-eso-10.1177_23969873211031753 for Differences in cerebral small vessel disease magnetic resonance imaging markers between lacunar stroke and non–Lobar intracerebral hemorrhage by Kim Wiegertjes, Michelle G Jansen, Wilmar MT Jolink, Marco Duering, Emma A Koemans, Floris HBM Schreuder, Anil M Tuladhar, Marieke JH Wermer, Catharina JM Klijn and Frank-Erik de Leeuw in European Stroke Journal

sj-pdf-3-eso-10.1177_23969873211031753 - Supplemental material for Differences in cerebral small vessel disease magnetic resonance imaging markers between lacunar stroke and non–Lobar intracerebral hemorrhage Click here for additional data file. Supplemental material, sj-pdf-3-eso-10.1177_23969873211031753 for Differences in cerebral small vessel disease magnetic resonance imaging markers between lacunar stroke and non–Lobar intracerebral hemorrhage by Kim Wiegertjes, Michelle G Jansen, Wilmar MT Jolink, Marco Duering, Emma A Koemans, Floris HBM Schreuder, Anil M Tuladhar, Marieke JH Wermer, Catharina JM Klijn and Frank-Erik de Leeuw in European Stroke Journal
