# Supplementary material for: Differences in cerebral small vessel disease magnetic resonance imaging markers between lacunar stroke and non–Lobar intracerebral hemorrhage
Source: Eur Stroke J. 2021 Aug 25;6(3):236–44. doi: 10.1177/23969873211031753 (PMC8564151; doi:10.1177/23969873211031753)
Supplement: sj-pdf-1-eso-10.1177_23969873211031753 - Supplemental material for Differences in cerebral small vessel disease magnetic resonance imaging markers between lacunar stroke and non–Lobar intracerebral hemorrhage [file sj-pdf-1-eso-10.1177_23969873211031753.pdf]

Supplemental material

Table 1. Overview of MRI parameters

	**RUN DMC**	**FETCH**		
	Nijmegen (n=82)	Nijmegen (n=20)	Utrecht (n=23)	Leiden (n=11)
MRI scanner	1.5 T Magnetom scanner, Siemens, Erlangen, Germany	3 T Magnetom Prisma Fit, Siemens, Erlangen, Germany	3 T unenhanced scanner, Philips Healthcare, Best, The Netherlands	3 T unenhanced scanner, Philips Healthcare, Best, The Netherlands
T1				
TR (ms)	2250	1900	7.9	9.8
TE (ms)	3.68	2.52	4.50	4.85
Voxel size (mm)	1.0 x 1.0 x 1.0	0.98 x 0.98 x 1.0	1.0 x 1.0 x 1.0	1.0 x 1.0 x 1.0
FLAIR				
TR (ms)	9000	9000	11000	11000
TE (ms)	84	87	125	120
Voxel size (mm)	1.2 x 1.0 x 6.0	0.60 x 0.60 x 5.0	0.96 x 0.96 x 3.0	0.96 x 0.96 x 5.0
Blood–sensitive sequence	T2*–weighted gradient echo	T2*–weighted gradient echo	T2*–weighted gradient echo	SWI
TR	800	27	1653	20
TE	26	20	20	40
Voxel size (mm)	1.3 x 1.0 x 5.0	0.98 x 0.98 x 3.0	0.96 x 0.96 x 3.0	0.96 x 0.96 x 3.0

Abbreviations: Magnetic resonance imaging parameters for RUN DMC and FETCH study. TR = repetition time; TE = echo time; FLAIR = fluid–attenuated recovery; SWI = susceptibility weighted image.

Figure 1. Overview of subject inclusion

Abbreviations: TIA = transient ischemic attack; ICH = intracerebral hemorrhage; IVH = intraventricular hemorrhage.

Figure 2. Voxel – based lesion – symptom mapping results for WMH frequency in MNI space

Voxel – wise lesion symptom mapping results, including the association between location of white matter hyperintensities and stroke subtype (lacunar stroke vs. non – lobar intracerebral hemorrhage), adjusted for age and total white matter hyperintensity volumes. The thresholded statistical map at a family – wise error rate of 5% has been superimposed on the MNI – 152 2mm template. We show voxels that are significantly more associated with non – lobar intracerebral hemorrhage in red, whereas voxels that were more associated with lacunar stroke are shown in blue.
