# Supplementary figures and images for: Differences in cerebral small vessel disease magnetic resonance imaging markers between lacunar stroke and non–Lobar intracerebral hemorrhage
Source: Eur Stroke J. 2021 Aug 25;6(3):236–44. doi: 10.1177/23969873211031753 (PMC8564151; doi:10.1177/23969873211031753)

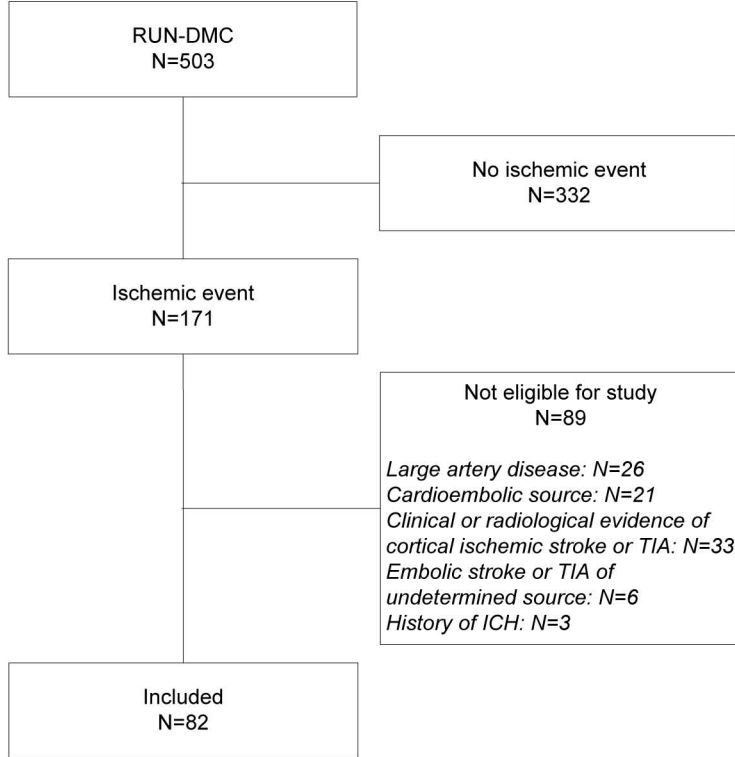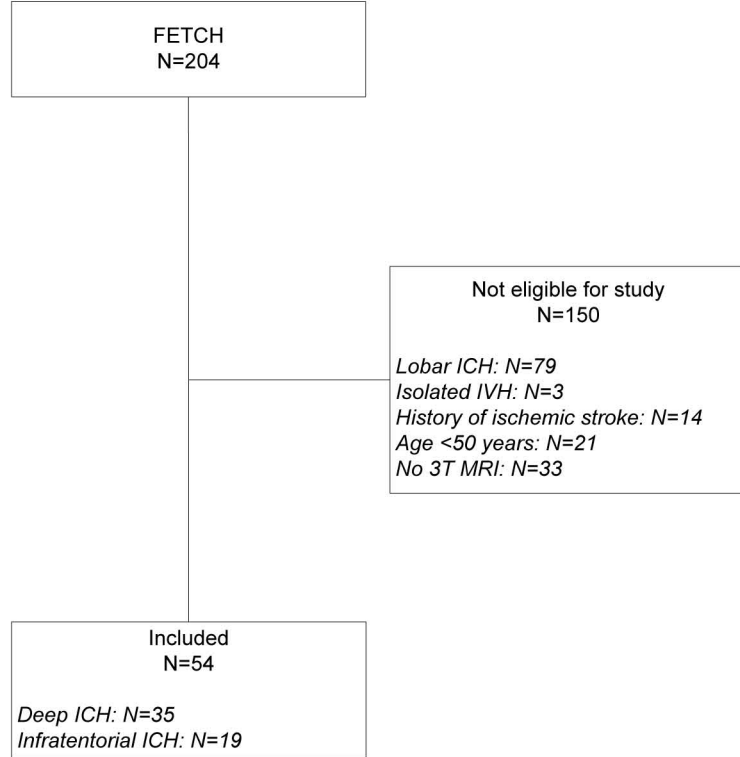

Supplement: sj-pdf-2-eso-10.1177_23969873211031753 - Supplemental material for Differences in cerebral small vessel disease magnetic resonance imaging markers between lacunar stroke and non–Lobar intracerebral hemorrhage [file sj-pdf-2-eso-10.1177_23969873211031753.pdf]

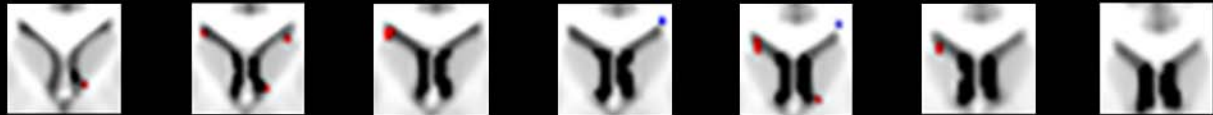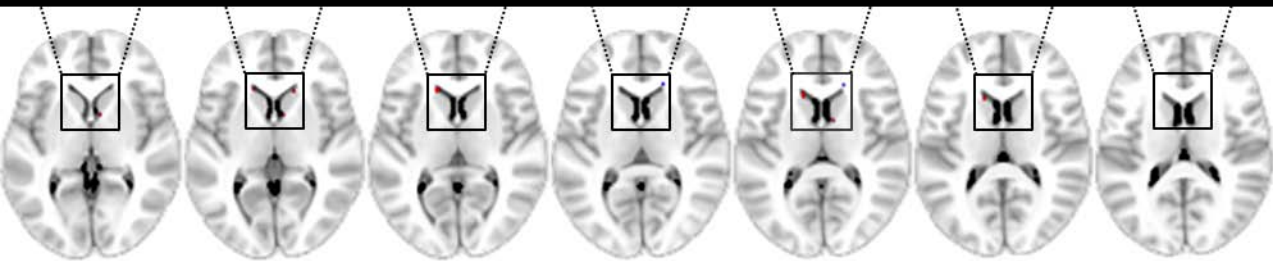

Supplement: sj-pdf-3-eso-10.1177_23969873211031753 - Supplemental material for Differences in cerebral small vessel disease magnetic resonance imaging markers between lacunar stroke and non–Lobar intracerebral hemorrhage [file sj-pdf-3-eso-10.1177_23969873211031753.pdf]
